# Iatrogenic Acute Aortic Insufficiency Following Mitral Valve Replacement

**DOI:** 10.7759/cureus.26570

**Published:** 2022-07-05

**Authors:** Abdalhai Alshoubi

**Affiliations:** 1 Anesthesia and Critical Care, St. Joseph’s Medical Center, Stockton, USA

**Keywords:** aortic valve, intraoperative transesophageal echocardiography, surgical complications, mitral valve surgery, iatrogenic aortic regurgitation

## Abstract

Iatrogenic acute aortic insufficiency after heart procedures is rare and it can happen secondary to cusp entrapment, tension, laceration, or perforation. The aortic valve is located anterior and to the right of the mitral valve, which makes it susceptible to damage during mitral valve replacement or repair. We report a 62-year-old male who developed acute aortic valve insufficiency following mitral valve replacement where using an intraoperative transesophageal echocardiogram (TEE) prompted early diagnosis and management. Late diagnosis is usually associated with increased morbidity and mortality. The aortic insufficiency resulted from entrapment of the aortic valve annulus due to suture misplacement at the commissure between the left and noncoronary cusp. This case report shines the light on the importance of a thorough intraoperative TEE during cardiac surgery to early diagnose and treat any complications.

## Introduction

Aortic regurgitation is a known complication after cardiac surgeries that are performed in close proximity of the aortic valve. This complication happened due to aortic annulus entrapment, cusp perforation, or injury. Suture-related aortic annulus entrapment is a rare event during mitral valve surgery.

This report illustrates intraoperative echocardiographic findings of iatrogenic aortic valve insufficiency following mitral valve replacement due to entrapment of the aortic valve annulus at the commissure between the left and noncoronary cusp.

## Case presentation

A 62-year-old male with a past medical history of hypertension, hyperlipidemia, chronic atrial fibrillation, severe coronary artery disease, and severe mitral valve insufficiency secondary to myxomatous degeneration and prolapse of both the anterior and posterior leaflets with cleft posterior leaflets between P1 and P2. The preoperative transthoracic echocardiogram showed a normal aortic valve with an ejection fraction (EF) of 60%. The patient underwent coronary artery bypass grafting x 3. The mitral valve was replaced using a 31-mm Mosaic valve (Medtronic, Minneapolis, MN) after resection of the anterior and posterior leaflets and placement of the sutures in a sequential fashion around the annulus.

**Figure 1:**
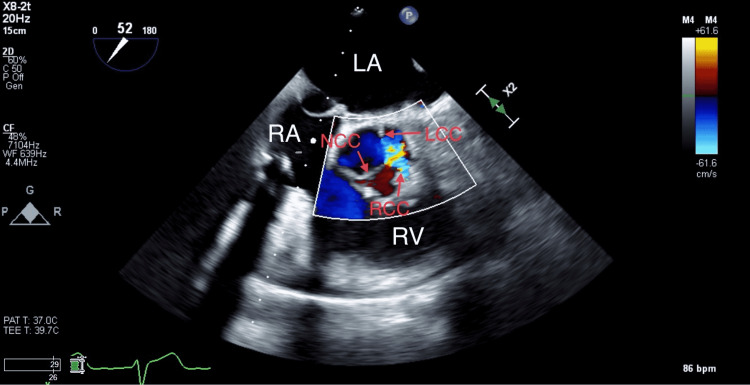
Mid-esophageal TEE aortic valve short-axis view showing aortic insufficiency due to aortic annulus entrapment. TEE: transesophageal echocardiogram, NCC: noncoronary cusp, LCC: left coronary cusp, RCC: right coronary cusp, LA: left atrium, RA: right atrium, and RV: right ventricle.

Following weaning off cardiopulmonary bypass (CPB), the transesophageal echocardiogram (TEE) showed a well-seated 31-mm Mosaic valve with no regurgitation or leak. On the other hand, a severe aortic insufficiency was noted (Figure 1).

**Figure 2:**
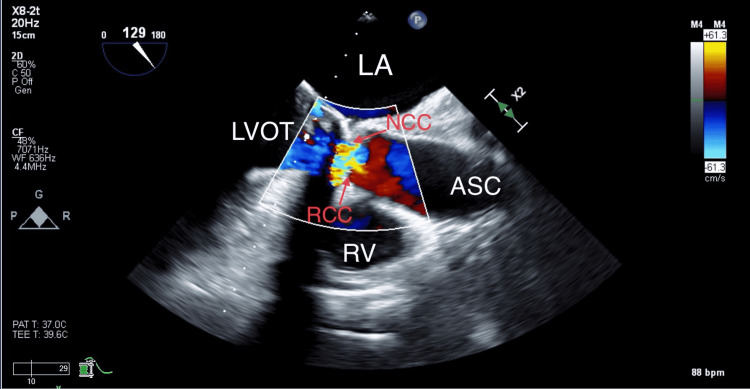
Mid-esophageal TEE aortic valve long-axis view showing aortic insufficiency due to aortic annulus entrapment. NCC: noncoronary cusp, RCC: right coronary cusp, LA: left atrium, LVOT: Left ventricular outflow tract, ASC: ascending aorta, and RV: right ventricle.

The mid-esophageal TEE long-axis view of the aortic valve showed aortic insufficiency due to aortic annulus entrapment preventing proper closure of the aortic cups (Figure 2).

**Figure 3:**
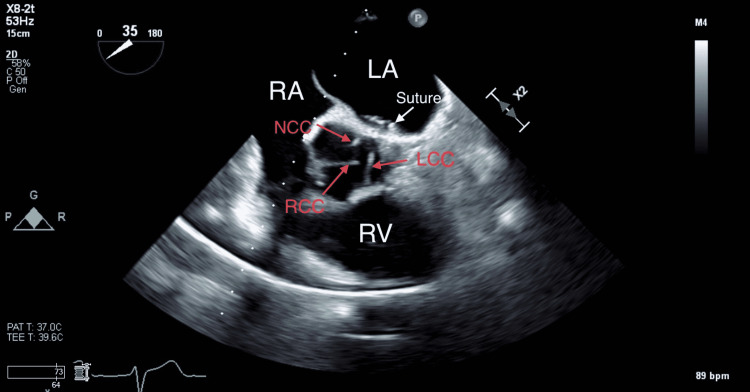
Mid-esophageal TEE aortic valve short-axis view showing aortic annulus entrapment due to suture placement between NCC and LCC. NCC: noncoronary cusp, LCC: left coronary cusp, RCC: right coronary cusp, LA: left atrium, RA: right atrium, and RV: right ventricle.

After reinstitution of CPB, we found out that one of the mitral valve sutures had tethered the aortic valve annulus at the commissure between the left and noncoronary cusp, which was cut. Reinforcement sutures were applied. There were no abnormalities with the mitral valve (Figure 3).

**Figure 4:**
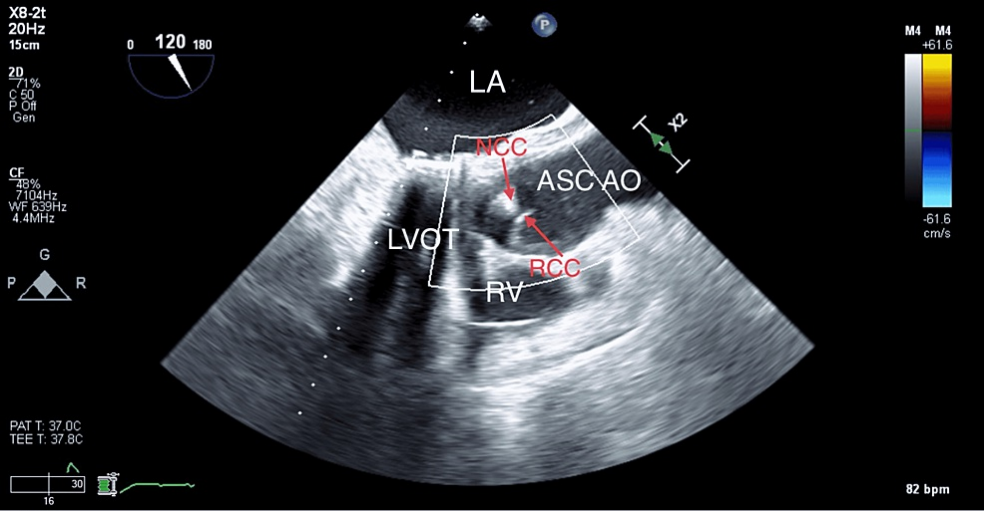
Postoperative mid-esophageal TEE aortic valve long-axis view showing normal aortic valve with no evidence of aortic insufficiency. NCC: noncoronary cusp, RCC: right coronary cusp, LA: left atrium, LVOT: left ventricular outflow tract, ASC AO: ascending aorta, and RV: right ventricle.

A repeat TEE showed a normal aortic valve with no evidence of any insufficiency (Figure 4).

Postoperatively, the patient was admitted to the intensive care unit, where he was extubated six hours later. Physical therapy started shortly after. On a postoperative day four, the patient was transferred to the ward and discharged home in stable condition on postoperative day eight.

## Discussion

Mechanical injury to the aortic valve cups and annulus during mitral valve replacement or repair is rare. These injuries are mostly related to cusp tear, perforation, or tethering and can affect any of the three cusps due to accidental suture placement or needle laceration in the setting of difficult visualization of the aortic valve during the left atrial approach for mitral surgeries [1,2]. Due to anatomical proximity, the most commonly affected cusps during mitral valve surgeries are the noncoronary and left coronary cusps; it rarely affects the right coronary cusp [3]. Aortic valve injury has also been reported following blunt chest trauma, percutaneous coronary intervention, Impella device placement, ventricular septal defect repair, atrial septal defect repair, and left ventricular septal myectomy [4,5].

TEE is the golden standard diagnostic test. However, mid-esophageal views might not be helpful due to the interference of the ultrasound artifacts created by the prosthetic valve. Adding three-dimensional (3D) TEE provides detailed imaging, which makes diagnosing valvular perforation more feasible [6]. The typical imaging findings using TEE are echo dropout in the body of the cusp and an eccentric regurgitant jet. The origin of regurgitant flow can be confirmed using a short-axis view and three-dimensional TEE [7].

The management depends on the mechanism of the injury and the severity. Mild aortic valve insufficiency can be managed medically. Moderate to severe aortic insufficiency due to perforated cusps is repaired with a pericardial patch or aortic valve replacement depending on the degree of the damage. Aortic cusp and annulus entrapment due to suture placement are treated by cutting the suture, as was the case in our patient.

## Conclusions

Our case emphasizes the importance of using intraoperative TEE for early diagnosis of iatrogenic acute severe aortic insufficiency after mitral valve replacement, which prompted early management. Late diagnosis is usually associated with increased morbidity and mortality.
